# Comparative performance of lung cancer risk models to define lung screening eligibility in the United Kingdom

**DOI:** 10.1038/s41416-021-01278-0

**Published:** 2021-04-12

**Authors:** Hilary A. Robbins, Karine Alcala, Anthony J. Swerdlow, Minouk J. Schoemaker, Nick Wareham, Ruth C. Travis, Philip A. J. Crosbie, Matthew Callister, David R. Baldwin, Rebecca Landy, Mattias Johansson

**Affiliations:** 1 International Agency for Research on Cancer, Lyon, France; 2 The Institute of Cancer Research, London, UK; 3 University of Cambridge, Cambridge, UK; 4 Cancer Epidemiology Unit, Nuffield Department of Population Health, University of Oxford, Oxford, UK; 5 University of Manchester, Manchester, UK; 6 Leeds Teaching Hospitals, Leeds, UK; 7 Nottingham University Hospitals and University of Nottingham, Nottingham, UK; 8 Division of Cancer Epidemiology and Genetics, Department of Health and Human Services, National Cancer Institute, National Institutes of Health, Bethesda, MD USA

**Keywords:** Epidemiology, Lung cancer, Lung cancer, Cancer epidemiology, Cancer screening

## Abstract

**Background:**

The National Health Service England (NHS) classifies individuals as eligible for lung cancer screening using two risk prediction models, PLCOm2012 and Liverpool Lung Project-v2 (LLPv2). However, no study has compared the performance of lung cancer risk models in the UK.

**Methods:**

We analysed current and former smokers aged 40–80 years in the UK Biobank (*N* = 217,199), EPIC-UK (*N* = 30,813), and Generations Study (*N* = 25,777). We quantified model calibration (ratio of expected to observed cases, *E*/*O*) and discrimination (AUC).

**Results:**

Risk discrimination in UK Biobank was best for the Lung Cancer Death Risk Assessment Tool (LCDRAT, AUC = 0.82, 95% CI = 0.81–0.84), followed by the LCRAT (AUC = 0.81, 95% CI = 0.79–0.82) and the Bach model (AUC = 0.80, 95% CI = 0.79–0.81). Results were similar in EPIC-UK and the Generations Study. All models overestimated risk in all cohorts, with *E*/*O* in UK Biobank ranging from 1.20 for LLPv3 (95% CI = 1.14–1.27) to 2.16 for LLPv2 (95% CI = 2.05–2.28). Overestimation increased with area-level socioeconomic status. In the combined cohorts, USPSTF 2013 criteria classified 50.7% of future cases as screening eligible. The LCDRAT and LCRAT identified 60.9%, followed by PLCOm2012 (58.3%), Bach (58.0%), LLPv3 (56.6%), and LLPv2 (53.7%).

**Conclusion:**

In UK cohorts, the ability of risk prediction models to classify future lung cancer cases as eligible for screening was best for LCDRAT/LCRAT, very good for PLCOm2012, and lowest for LLPv2. Our results highlight the importance of validating prediction tools in specific countries.

## Background

Lung cancer is the leading cause of cancer death worldwide.^1,2^ Two large, randomised trials have now demonstrated that screening by low-dose computed tomography (LDCT) can reduce mortality from lung cancer among people with a heavy smoking history. Lung cancer mortality was reduced by 20% over 5 years in the USA National Lung Screening Trial (NLST) with 3 annual LDCT screens^3^ and by 24% (men) and 33% (women) over 10 years in the Dutch-Belgian NELSON trial with 4 LDCT screens over 5.5 years.^4^

The USA issued a national recommendation for lung screening in 2014.^5^ In the United Kingdom, there have been several successful pilot studies, including the Manchester Lung Health Checks^6,7^ and the Liverpool Healthy Lung Programme.^8^ Compared with the implementation of lung screening in the USA, the UK has often been more successful in terms of overall uptake and engagement of populations with low socioeconomic status (SES),^6,9,10^ and lung cancer detection rates have often exceeded those in the NLST.^3,6,11,12^ Building on this success, the National Health Service (NHS) England is implementing a £70 million programme of “Targeted Lung Health Checks” in 10 areas with high lung cancer mortality.^13,14^

In the USA, the US Preventive Services Task Force (USPSTF) guidelines use categorical criteria to determine who is eligible for screening. Eligibility by the 2013 guideline required age 55–80 years, at least 30 pack-years smoked, and for former smokers, no more than 15 years since quitting.^5^ The 2020 draft guideline expands eligibility by lowering the age-to-start from 55 to 50 years, and lowering the pack-year threshold from 30 to 20 pack-years.^15^ However, secondary analyses of the NLST demonstrated that lung screening may be more efficient and cost-effective when eligibility is based on individual lung cancer risk, estimated using a continuous risk prediction model.^16–19^ Lung screening in the UK was implemented using individual risk-based eligibility from the beginning, and the NHS England protocol specifies that individuals aged 55–74 years can be screened if their lung cancer risk exceeds 1.51% by the PLCOm2012 model (6-year risk) or 2.5% by the Liverpool Lung Project version 2 (LLPv2) model (5-year risk).^14^

The choice of which risk model to use for screening eligibility is important. Poor model discrimination or calibration can reduce the efficiency and cost-effectiveness of screening and even lead to net harm if models select individuals who are unlikely to benefit from screening. Risk models differ in the variables that they include; for example, the LLP/LLPv2/LLP model version 3 (LLPv3) models include only one measure of smoking (duration), whereas the Lung Cancer Death Risk Assessment Tool (LCDRAT) includes smoking duration, pack-years, quit-years, and intensity.^18,20^ Most models, including PLCOm2012 and LCDRAT, were developed using USA data, whereas the LLP/LLPv2/LLPv3 models were developed in the UK.^17,18,20^ Although both PLCOm2012 and LLPv2 have been implemented successfully in screening studies, the absence of outcome data on individuals who were not eligible (and thus not screened) has precluded evaluation of whether either of these is the optimal model.^6,11^ Several models have been evaluated in population cohort studies in the USA,^21,22^ but non-USA evaluations are scarce,^23,24^ and none include data from UK cohorts.

Here we performed a comparative evaluation of lung cancer risk models to define lung screening eligibility in the UK. We analysed 3 cohort studies to quantify the calibration and discrimination of risk models and then compared their ability to classify future lung cancer cases into a group defined as eligible for screening.

## Methods

We analysed longitudinal data from the UK Biobank, European Prospective Investigation into Cancer and Nutrition (EPIC)-UK, and Generations Study cohorts. The UK Biobank is a prospective cohort study of 500,000 people aged 40–72 years at recruitment (2006–2010).^25^ EPIC-UK recruited participants aged 45–74 years in Cambridge and aged ≥20 years in Oxford during 1993–2000.^26^ EPIC-Cambridge used population-based recruitment of patients of general practitioners, while EPIC-Oxford was comprised of both population-based recruitment and a subset targeted at “health conscious” individuals. Finally, the Generations Study recruited 112,000 women aged ≥16 years during 2003–2011, of whom about one-third had a mother, daughter, or sister also participating in the study.^27^ In all cohorts, cancer and death ascertainment relied on registry linkages at minimum, sometimes with additional active follow-up.^25–27^

From all participants in these cohorts, we restricted to those known to be current or former smokers who were aged 40–80 years at enrolment, including 217,199 in UK Biobank, 30,813 in EPIC-UK, and 25,777 in the Generations Study (total *N* = 273,789). Never smokers and participants with unknown smoking status were excluded. After these restrictions, substantial amounts of missing data were present for some variables in some cohorts, such as 31% missing smoking intensity (cigarettes per day) in UK Biobank. Missing data were handled using various approaches within the framework of multiple imputation (see Supplement). Among participants who were alive and free of lung cancer at the end of follow-up (i.e. in whom future lung cancer status would be unknown), follow-up time was at least 6 years for all participants in UK Biobank and EPIC-UK and for 88% in the Generations Study.

We evaluated 8 lung cancer risk models. These included the PLCOm2012 and LLPv2 models, which are proposed for use in selecting screening participants in the NHS protocol.^11,14,17,20^ We also evaluated the Bach model,^28^ the LCDRAT,^18^ the Lung Cancer Risk Assessment Tool (LCRAT),^18^ the original LLP model,^20^ the LLPv3,^29^ and the Hoggart model.^30^ Each of these models is either a USA-based model that performs well in USA data (Bach, LCDRAT, LCRAT, PLCOm2012)^21^ or a European model whose performance in European data is unknown (LLP, LLPv2, LLPv3, Hoggart). Risk thresholds above which screening can be offered have been proposed for LCRAT and LCDRAT,^19,31^ and LLPv3,^29^ in addition to PLCOm2012 and LLPv2.

Risk estimates for the LCRAT (5-year time horizon), LCDRAT (5-year), and Hoggart (1-year) models were generated using the lcmodels package in R.^32^ Estimates for the Bach model used code adapted from lcmodels to reduce the time horizon to 5 years. Estimates for PLCOm2012 (6-year time horizon), LLPv3 (5-year), LLPv2 (5-year), and LLP (5-year) were calculated directly.^17,20^ We present results for two models in Supplementary Table 1 and do not include them in discussions below, due to redundancy with LLPv2/LLPv3 (LLP) and very high overestimation of risk (Hoggart). We present results for LLPv2 in the main manuscript, even though it may be eventually replaced by LLPv3, because LLPv2 is listed in the NHS England protocol.

We calculated calibration as the ratio of expected to observed (*E*/*O*) lung cancer cases or deaths, overall and in subgroups. We quantified discrimination using the area under the receiver-operating curve (AUC) statistic. The 95% confidence intervals (CIs) for calibration and discrimination statistics account for within and between imputation variance.^21^

## Results

Among the 217,199 current or former smokers aged 40–80 years in UK Biobank, 1265 lung cancer cases were diagnosed within 5 years of enrolment, and 700 lung cancer deaths occurred in this period (Table 1). In EPIC-UK, 156 lung cancers and 100 lung cancer deaths occurred over 5 years among 30,813 participants, and in the Generations Study, 53 lung cancers and 26 lung cancer deaths occurred over 5 years among 25,777 participants. Distributions of demographic and smoking variables differed across cohorts.

**Table 1:** Characteristics of current and former smokers in UK Biobank, EPIC-UK, and the Generations Study.

Characteristic	UK Biobank	EPIC-UK	Generations Study
All participants	217,199	30,813	25,777
Lung cancer cases			
Within 1 year of enrolment	194	23	5
Within 2 years of enrolment	420	58	16
Within 5 years of enrolment	1265	156	53
Within 6 years of enrolment	1461	196	68
Lung cancer deaths			
Within 5 years of enrolment	700	100	26
Eligible by USPSTF 2013 criteria^a^	26,644 (12.3%)	2484 (8.1%)	1110 (4.3%)
Eligible by USPSTF 2020 criteria^a^	45,065 (20.7%)	5179 (16.8%)	2994 (11.6%)
Lung cancer cases within 5 years of enrolment			
Among USPSTF 2013 eligible individuals^a^	616 (48.7%)	59 (37.8%)	17 (32.1%)
Among USPSTF 2020 eligible individuals^a^	805 (63.6%)	75 (48.1%)	24 (45.3%)
Age at recruitment, years			
40–44	19,881 (9.2%)	3508 (11.4%)	3815 (14.8%)
45–49	24,954 (11.5%)	5782 (18.8%)	4124 (16.0%)
50–54	30,633 (14.1%)	5277 (17.1%)	4773 (18.5%)
55–59	39,632 (18.2%)	4302 (14.0%)	5417 (21.0%)
60–64	56,626 (26.1%)	3813 (12.4%)	4481 (17.4%)
65–69	44,389 (20.4%)	3877 (12.6%)	2067 (8.0%)
70–74	1084 (0.5%)	3268 (10.6%)	799 (3.1%)
75–80	0 (0.0%)	986 (3.2%)	301 (1.2%)
Sex			
Male	108,043 (49.7%)	13,191 (42.8%)	0 (0.0%)
Female	109,156 (50.3%)	17,622 (57.2%)	25,777 (100.0%)
Smoking status			
Current	50,151 (23.1%)	6721 (21.8%)	4116 (16.0%)
Former, 0–4 quit years	15,094 (6.9%)	2227 (7.2%)	2057 (8.0%)
Former, 5–9 quit years	14,431 (6.6%)	2583 (8.4%)	1972 (7.7%)
Former, 10–14 quit years	12,513 (5.8%)	3374 (10.9%)	2259 (8.8%)
Former, 15–19 quit years	12,753 (5.9%)	3590 (11.7%)	2772 (10.8%)
Former, ≥20 quit years	54,610 (25.1%)	10,025 (32.5%)	11,579 (44.9%)
Former, Unknown	57,647 (26.5%)	2293 (7.4%)	1022 (4.0%)
Cigarettes smoked per day			
≤10	43,870 (20.2%)	9845 (32.0%)	11,223 (43.5%)
11–19	31,488 (14.5%)	8249 (26.8%)	7278 (28.2%)
20–29	55,168 (25.4%)	5427 (17.6%)	2905 (11.3%)
30–39	11,468 (5.3%)	711 (2.3%)	408 (1.6%)
≥40	8619 (4.0%)	435 (1.4%)	125 (0.5%)
Unknown	66,586 (30.7%)	6146 (19.9%)	3838 (14.9%)
Educational level			
Less than secondary	44,950 (20.7%)	6248 (20.3%)	6 (0.0%)
Secondary degree	58,098 (26.7%)	12,031 (39.0%)	10,947 (42.5%)
Some post-secondary training	33,688 (15.5%)	NA	NA
Some university	16,126 (7.4%)	NA	7996 (31.0%)
University graduate	60,363 (27.8%)	6369 (20.7%)	6223 (24.1%)
Unknown	3974 (1.8%)	6165 (20.0%)	605 (2.3%)
Body mass index			
Underweight	1100 (0.5%)	356 (1.2%)	167 (0.6%)
Normal weight	64,430 (29.7%)	14,776 (48.0%)	12,220 (47.4%)
Overweight	93,806 (43.2%)	11,927 (38.7%)	8658 (33.6%)
Obese	56,651 (26.1%)	3754 (12.2%)	4233 (16.4%)
Unknown	1212 (0.6%)	0 (0.0%)	499 (1.9%)
Personal history of cancer	10,651 (4.9%)	1123 (3.6%)	1722 (6.7%)
First-degree family history of lung cancer	18,039 (8.3%)	Missing	Missing
COPD or emphysema	4046 (1.9%)	Missing	Missing
Prior pneumonia	1346 (0.6%)	Missing	Missing
Asbestos exposure	Missing	2167 (7.0%)	Missing

Table 1 shows data prior to imputation of missing data. UK educational categories were mapped to USA categories as described in the Supplement. Body mass index categories were defined as follows: <18.5 underweight, 18.5–24.9 normal weight, 25–29.9 overweight, and ≥30 obese. “Asbestos exposure” reflects self-reported occupational asbestos exposure.

*COPD* chronic obstructive pulmonary disease, *NA* not applicable.

^a^Eligibility by the US Preventive Services Task Force (USPSTF) 2013 criteria requires age 55–80 years, at least 30 pack-years, and no more than 15 quit-years. Eligibility by the draft USPSTF 2020 criteria requires age 50–80 years, at least 20 pack-years, and no more than 15 quit-years.

**Fig. 1:**
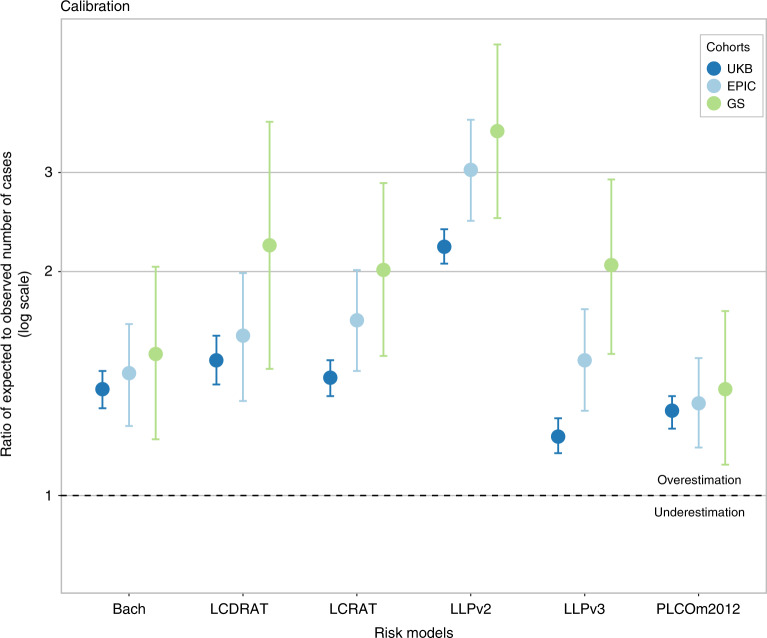
Calibration of lung cancer risk models in the UK Biobank, EPIC-UK, and Generations Study cohorts, as measured by the ratio of expected to observed cases. UKB UK Biobank, GS Generations Study. Estimates for UK Biobank also appear in Table 2 and Supplementary Table 3.

Calibration estimates for all risk models were >1 in all cohorts, indicating that the models predicted more lung cancer cases (or for LCDRAT, lung cancer deaths) than were observed over the time period specified by the model (Fig. 1). The extent of overestimation of risks (and therefore poorest calibration) was the highest in the Generations Study and the lowest in UK Biobank for each model. Across the risk models, in UK Biobank, LLPv3 was best calibrated (*E*/*O* = 1.20, 95% CI = 1.14–1.27), followed by PLCOm2012 (*E*/*O* = 1.30, 95% CI 1.23–1.36), the Bach model (*E*/*O* = 1.39, 95% CI 1.31–1.47), LCRAT (*E*/*O* = 1.44, 95% CI 1.36–1.52), and LCDRAT (*E*/*O* = 1.52, 95% CI 1.41–1.64). Overestimation was the highest for LLPv2 (*E*/*O* = 2.16, 95% CI 2.05–2.28). The order in which models were ranked in EPIC-UK and the Generations Study was similar to UK Biobank, but *E*/*O* statistics were higher.

**Fig. 2:**
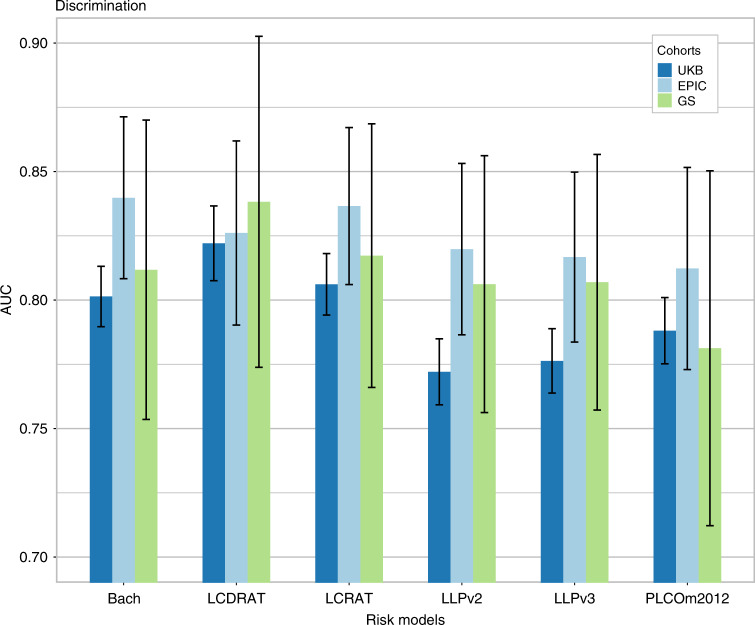
Discrimination of lung cancer risk models in the UK Biobank, EPIC-UK, and Generations Study cohorts, as measured by the area under the ROC curve (AUC). UKB UK Biobank, GS Generations Study. Estimates for UK Biobank also appear in Supplementary Tables 2 and 3.

Differences in discrimination estimates (AUCs) across models were modest. In UK Biobank, discrimination was the highest for LCDRAT (AUC = 0.82, 95% CI 0.81–0.84) and the lowest for LLPv2 (AUC = 0.77, 95% CI 0.76–0.78) (Fig. 2). However, this ordering differed in EPIC-UK, ranging from AUC = 0.84 for the Bach model (95% CI 0.81–0.87) to 0.81 for PLCOm2012 (95% CI 0.77–0.85). Discrimination in the Generations Study ranged from AUC = 0.84 for LCDRAT (95% CI 0.77–0.90) to 0.78 for PLCOm2012 (95% CI 0.71–0.85).

To further investigate model overestimation, we calculated *E*/*O* estimates stratified by demographic and smoking characteristics in UK Biobank (Table 2). Analogous estimates for discrimination (stratified AUCs) are presented in Supplementary Table 2. For all models, there was a strong positive relationship between model overestimation and SES, which was measured by the area-level Townsend deprivation index. For example, for LCDRAT, *E*/*O* statistics across SES quartiles were 2.03 (highest SES), 1.95, 1.61, and 1.26 (lowest SES). Patterns for other characteristics differed across models, though frequent patterns included more overestimation in men than in women, in former smokers than in current smokers, and at the extremes of age (40s and 70s). When stratifying by quintiles of predicted risk (Supplementary Fig. 1), PLCOm2012 substantially underestimated risk in the lowest-risk quintile while modestly overestimating risk in the upper categories. Overestimation tended to be higher at higher risks for LLPv2, LLPv3, and Bach, while it was higher at lower risks for LCDRAT and LCRAT.

**Table 2:** Calibration estimates for lung cancer risk models in the subgroups of UK Biobank, as measured by the ratio of expected to observed cases.

	Calibration estimate (expected/observed) by risk model					
	Bach	LCDRAT	LCRAT	LLPv2	LLPv3	PLCOm2012
All participants	1.39	1.52	1.44	2.16	1.20	1.30
Sex						
Male	1.50	1.56	1.50	2.46	1.20	1.36
Female	1.25	1.47	1.37	1.81	1.20	1.23
Age, years						
40–49	1.69	2.16	2.51	1.31	0.73	2.20
50–59	1.36	1.32	1.47	1.73	1.00	1.28
60–69	1.38	1.56	1.38	2.32	1.28	1.25
70–74	1.89	2.61	1.93	3.52	1.92	2.12
Area-level SES						
Q1 (highest SES)	1.89	2.03	1.93	3.06	1.69	1.64
Q2	1.79	1.95	1.84	2.78	1.54	1.57
Q3	1.51	1.61	1.57	2.20	1.22	1.39
Q4 (lowest SES)	1.14	1.26	1.21	1.49	0.82	1.06
Smoking status						
Current	1.28	1.39	1.44	1.60	0.88	1.10
Former	1.48	1.65	1.44	2.64	1.47	1.47
Smoking intensity						
≤10 CPD	1.17	1.40	1.50	2.96	1.70	0.65
11–29 CPD	1.43	1.52	1.41	2.09	1.16	1.44
≥30 CPD	1.48	1.64	1.48	1.63	0.86	1.48
Education						
Less than secondary	1.10	1.31	1.20	1.64	0.91	1.10
Secondary degree	1.58	1.82	1.70	2.41	1.36	1.51
Some post-secondary training	1.45	1.52	1.48	2.30	1.29	1.33
Some university	1.42	1.21	1.20	2.13	1.88	1.10
University graduate	1.97	2.01	1.96	3.39	1.13	1.71
Body mass index						
Underweight	0.98	1.52	1.64	1.40	0.84	1.20
Normal weight	1.09	1.41	1.36	1.76	1.00	1.17
Overweight	1.50	1.61	1.55	2.41	1.31	1.39
Obese	1.59	1.52	1.38	2.31	1.28	1.31

Estimates are provided for UK Biobank only due to the small size of the other cohorts. SES is measured using the Townsend deprivation index, an area-level measure that is applied to individuals based on their place of residence. Quartiles of the Townsend deprivation index were defined such that UK Biobank participants were divided equally, using the following cutpoints: −6.26 (minimum), −3.42, −1.75, 1.21, 11.0 (maximum). Body mass index categories were defined as follows: <18.5 underweight, 18.5–24.9 normal weight, 25–29.9 overweight, and ≥30 obese.

*CPD* cigarettes per day, *SES* socioeconomic status.

Table 3 considers the hypothetical impact of using each risk model to determine who is screening eligible in the combined population of UK Biobank, EPIC-UK, and the Generations Study. In the combined population, after imputing missing data, 15.0% of individuals were eligible for screening by USPSTF 2013 criteria (age 55–80 years, at least 30 pack-years, no more than 15 quit-years). The thresholds that would screen the same number of individuals using risk-based eligibility were 0.8% for LCDRAT (5-year lung cancer death risk), 1.4% for LCRAT (5-year lung cancer risk), 1.5% for PLCOm2012 (6-year risk), 1.6% for Bach (5-year risk), 1.3% for LLPv3 (5-year risk), and 2.3% for LLPv2 (5-year risk).

**Table 3:** Performance of lung cancer risk models for defining lung cancer screening eligibility among current and former smokers in the combined UK Biobank, EPIC-UK, and Generations Study cohorts.

Risk model	Threshold to screen the same number of participants as USPSTF guidelines	Lung cancer cases eligible for screening over 5 years	Lung cancer deaths eligible for screening over 5 years
USPSTF 2013 guidelines (age 55–80 years, at least 30 pack-years, no more than 15 quit-years)			
Total	NA	1474 (100%)	826 (100%)
USPSTF criteria	NA	747 (50.7%)	415 (50.2%)
LCDRAT	0.8% 5-year risk	897 (60.9%)	522 (63.2%)
LCRAT	1.4% 5-year risk	897 (60.9%)	519 (62.8%)
PLCOm2012	1.5% 6-year risk	859 (58.3%)	490 (59.3%)
Bach	1.6% 5-year risk	855 (58.0%)	501 (60.7%)
LLPv3	1.3% 5-year risk	835 (56.6%)	489 (59.2%)
LLPv2	2.3% 5-year risk	791 (53.7%)	465 (56.3%)
USPSTF 2020 guidelines (age 50–80 years, at least 20 pack-years, no more than 15 quit-years)			
Total	NA	1474 (100%)	826 (100%)
USPSTF criteria	NA	979 (66.4%)	560 (67.8%)
LCDRAT	0.4% 5-year risk	1135 (77.0%)	655 (79.3%)
LCRAT	0.8% 5-year risk	1101 (74.7%)	637 (77.1%)
PLCOm2012	0.8% 6-year risk	1109 (75.2%)	629 (76.2%)
Bach	0.8% 5-year risk	1131 (76.7%)	652 (78.9%)
LLPv3	0.7% 5-year risk	1039 (70.5%)	604 (73.1%)
LLPv2	1.3% 5-year risk	1020 (69.2%)	590 (71.4%)

Models are listed in order of their performance for identifying future lung cancer cases (USPSTF 2013). “Risk” refers to lung cancer death risk for LCDRAT and to lung cancer risk for all other models. Results are based on a combined data set, which uses a single imputation for missing data. USPSTF 2013 criteria classify 41,107 (15.0%) current and former smokers in the 3 cohorts combined as screening eligible, and USPSTF 2020 criteria classify 71,387 (26.1%) as eligible. These percentages differ from Table 1 as they are calculated after imputation, whereas Table 1 omits missing data.

*NA* not applicable.

Using USPSTF 2013 guidelines to define screening eligibility, 50.7% of future lung cancer cases (*N* = 747) would be classified as eligible for screening (Table 3). Similarly, 50.2% of future lung cancer deaths (*N* = 415) over 5 years would be screening eligible, among which some fraction could be prevented by earlier detection. Applying risk models and the thresholds described above to define the screened population identified higher proportions of future cases: the LCDRAT and LCRAT identified the highest proportion of future cases as screening eligible (each 60.9%, *N* = 897), followed by PLCOm2012 (58.3%, *N* = 859), Bach (58.0%, *N* = 855), LLPv3 (56.6%, *N* = 835), and LLPv2 (53.7%, *N* = 791). For lung cancer deaths, the ranking of models was similar, with LCDRAT identifying 63.2% of lung cancer deaths as screening eligible (*N* = 522), followed by LCRAT (62.8%, *N* = 519), Bach (60.7%, *N* = 501), PLCOm2012 (59.3%, *N* = 490), LLPv3 (59.2%, *N* = 489), and LLPv2 (56.3%, *N* = 465).

Applying USPSTF 2020 criteria (age 50–80 years, at least 20 pack-years, no more than 15 quit-years) yielded lower risk thresholds for screening and higher percentages of cases classified as screening eligible, as expected (Table 3). USPSTF 2020 criteria identified 26.1% of participants and 66.4% of future lung cancer cases as screening eligible. If risk models also screened the 26.1% highest-risk participants, LCDRAT would identify 77.0% of cases at a threshold of 0.4% 5-year risk (*N* = 1135) followed by Bach (76.7%, *N* = 1131 at 0.8% 5-year risk), PLCOm2012 (75.2%, *N* = 1109 at 0.8% 6-year risk), LCRAT (74.7%, *N* = 1101 at 0.8% 5-year risk), LLPv3 (70.5%, *N* = 1039 at 0.7% 5-year risk), and LLPv2 (69.2%, *N* = 1020 at 1.3% 5-year risk). Results for lung cancer deaths were similar.

We analysed individuals aged 40–80 years, but the NHS England protocol restricts eligibility to ages 55–74 years. When we repeated our analysis after restricting to individuals aged 55–74 years in UK Biobank (n.b. there were no participants in UK Biobank aged >74 years), AUCs decreased as expected due to the loss in prediction derived from age variation. However, the rank order of AUCs and the calibration results were not affected (Table 4).

**Table 4:** Calibration and discrimination estimates for lung cancer risk models in UK Biobank current and former smoking participants, restricted to ages 55–74 years.

	Lung cancer risk model					
	Bach	LCDRAT	LCRAT	LLPv2	LLPv3	PLCOm2012
Calibration (*E*/*O*)						
Among age 40–74 years	1.39 (1.31–1.47)	1.52 (1.41–1.64)	1.44 (1.36–1.52)	2.16 (2.05–2.28)	1.20 (1.14–1.27)	1.30 (1.23–1.36)
Among age 55–74 years	1.38 (1.30–1.47)	1.52 (1.41–1.64)	1.40 (1.32–1.48)	2.25 (2.13–2.39)	1.25 (1.18–1.33)	1.26 (1.19–1.33)
Discrimination (AUC)						
Among age 40–74 years	0.80 (0.79–0.81)	0.82 (0.81–0.84)	0.81 (0.79–0.82)	0.77 (0.76–0.78)	0.78 (0.76–0.79)	0.79 (0.78–0.80)
Among age 55–74 years	0.76 (0.75–0.78)	0.79 (0.77–0.81)	0.77 (0.76–0.79)	0.73 (0.72–0.75)	0.74 (0.73–0.75)	0.75 (0.74–0.77)

The *E*/*O* statistic is the ratio of cases expected (predicted by the model) to the cases observed. AUC is the area under the receiver operating curve. The estimates for ages 40–74 years correspond with the primary estimates presented in Fig. 1 (n.b. there were no participants in UK Biobank aged >74 years).

Supplementary Table 3 describes the characteristics of lung cancer cases that are not identified as screening eligible (are “missed”) by USPSTF 2013, USPSTF 2020, and each risk model at the risk thresholds identified in Table 3. Compared with USPSTF 2013, the cases missed by risk models, while fewer in number, were more commonly former smokers (62% of cases missed by USPSTF vs. 69–80% for risk models). They also tended to be slightly younger (median age at baseline 63 years for cases missed by USPSTF vs. 60–61 years for risk models) and slightly more frequently female (53% of cases missed by USPSTF vs. 54–57% for risk models). Patterns using thresholds based on USPTF 2020 were similar or more pronounced.

## Discussion

Lung cancer screening has the potential to substantially reduce lung cancer mortality among people with a heavy smoking history. In the United Kingdom, the success of the Targeted Lung Health Checks will depend partially on whether the programme can be implemented efficiently and cost-effectively. The protocol currently recommends the use of the PLCOm2012 and LLPv2 risk models to identify screening-eligible individuals.^14^ In this study, we compared the performance of these two models along with others that have performed well in other high-income settings. We found that the LLPv2 model had worst calibration and classified the lowest proportion of future lung cancer cases as eligible for screening. The PLCOm2012 model had better calibration, though all models predicted more cases than were observed. The LCDRAT was able to classify the highest proportion of future lung cancer cases as eligible for screening, with very good performance also observed for the LCRAT, PLCOm2012, and Bach models.

The models evaluated in our study were previously validated in multiple USA cohorts, including the NIH-AARP and CPS-II,^21^ as well as the NLST and PLCO trials.^22^ Taken together, these studies showed good calibration for the Bach model, LCRAT, LCDRAT, and PLCOm2012 but overestimation of risks for the LLP model. In our study, all models overestimated risks; the extent was greatest for LLPv2. For discrimination, prior results in NIH-AARP and CPS-II showed best performance for LCDRAT, followed sequentially by LCRAT, PLCOm2012, Bach, and LLP.^21^ The study analysing PLCO and NLST found higher discrimination for PLCOm2012 and Bach compared with LLP.^22^ The order in which models ranked in our study was similar, with best performance for the LCDRAT, LCRAT, Bach, and PLCOm2012 models. The likely explanation for inferior discrimination of the LLP/LLPv2/LLPv3 models is that they incorporate only smoking duration (omitting intensity and quit-years) and use categorical instead of continuous parameterisations of age and smoking.^20^ Overall, the magnitude of AUCs in our study (often exceeding 0.80) was higher than in prior reports (typically ranging from 0.75 to 0.80).^21,22^ This is likely caused by a wider age distribution in our analysis, which included more younger individuals.

In UK screening studies, lung cancer detection rates have commonly been higher than in the NLST.^3,6,7,11^ Lung cancer detection over 2 screens was 4.5% in the Manchester Lung Health Checks, compared with 1.7% in NLST.^3,6,7^ Detection rates in single-screen UK studies are commonly approximately 2%.^11,12^ These observations might have been taken as evidence that USA-based lung cancer risk models would predict too few cases in UK populations, but we found the opposite result. Unlike the screening studies, which commonly comprised individuals living in low-SES communities,^6,11,12^ the research cohorts we analysed have overrepresentation of high-SES individuals and are likely influenced by “healthy volunteer” effects. In UK Biobank, all-cause mortality among 70–74-year-olds is half that in the general population (although the difference in cancer incidence is smaller).^33^ EPIC-Oxford is partially comprised of “health-conscious” individuals. The Generations Study is a volunteer cohort with recruitment based on engagement in a health issue (finding the causes of breast cancer), and cancer incidence is estimated to be 16% lower than in the general UK population (unpublished data). These influences, taken together with the overrepresentation of high-SES individuals in whom overestimation is highest, suggest that model calibration would be better in the overall UK population of ever-smokers than in the research cohorts we analysed.

There is a troubling consequence to the correlation between risk model overestimation and SES. Considering four individuals with the same true risk of lung cancer, one in each SES quartile, the individual with the highest SES will have her risk overestimated the most and will be most likely to be classified as eligible for screening. This could exacerbate disparities in lung screening.^34^ Our findings suggest that there are factors related to SES that increase lung cancer risk but are not captured by the variables in risk models or are related to differential measurement error for the variables in risk models. Further, the effects of USA educational and ethnicity categories on lung cancer risk are unlikely to align with these effects in the UK. Any future efforts to develop risk models for use in lung screening in the UK should focus carefully on the role of SES and the accurate estimation of risk within subgroups.

The choice of what risk threshold to use for screening eligibility depends on multiple factors, including the accepted trade-off of benefits and harms and the capacity of the health system. We did not address these issues here, but we did identify thresholds that would classify the same number of individuals as screening eligible as USPSTF criteria. The thresholds selected by this approach, when considering USPSTF 2013 criteria, aligned with those already proposed for the PLCOm2012 (1.5% in our study vs. 1.51% in the NHS protocol) and the LLPv2 (2.3% in our study vs. 2.5% in the NHS protocol).^14^ There was a larger difference between the threshold we identified for LCDRAT (0.8% 5-year lung cancer death risk) and previously proposed thresholds (1.2, 1.33, 1.7%).^19,31^ Thresholds identified based on USPSTF 2020 criteria, which broadened eligibility substantially, were much lower, and it is not clear whether all individuals meeting these thresholds would have a favourable trade-off of screening benefits and harms.

Important limitations of risk-based eligibility for lung screening are receiving increased recognition. Risk models preferentially select older individuals, reducing life-years gained and cost-effectiveness, as well as individuals with comorbidities such as COPD who may have lower screening benefits.^19,35–37^ To address these issues, a model to define eligibility based on predicted life-years gained from screening has been proposed,^37^ which incorporates LCDRAT and an additional prediction model for overall mortality. Important evidence for the comparative performance of PLCOm2012 and LLPv2 will be provided by the Yorkshire Lung Screening Trial, which is enrolling participants based on eligibility by either model to compare their performance directly.^38^ The trial is also collecting sufficient information to validate the LCRAT, LCDRAT, and Bach models retrospectively.

Our study has important limitations that result from its approach of analysing cohort data. Our findings cannot be assumed to be nationally representative for the UK, though the rank-order performance of lung cancer risk prediction models is likely generalisable. There was also a substantial amount of missing data for key smoking variables, which we handled by multiple imputation. For comparison, we calculated *E*/*O* statistics and AUCs using the subset of individuals in UK Biobank who had complete data on required variables (63% of the cohort). The degree of overestimation was reduced for all models (Supplementary Fig. 2), while AUCs were not affected (Supplementary Fig. 3). By contrast, our approach of analysing multiple cohorts is a strength, because it allows for evaluating whether results are consistent across studies. Inclusion of the Generations Study, despite its small size, is important due to the underrepresentation of women in the European screening trial literature. Women in the Generations Study had lower smoking intensity and longer periods of cessation compared with participants in the other studies, highlighting potential equity issues around screening eligibility.

The question of which risk model optimally defines screening eligibility is somewhat distinct from the question of which model can most practically be implemented. Web-based tools are available for some risk models, including the Risk-based NLST Outcomes Tool for LCDRAT and LCRAT^39^ and MyLungRisk for LLPv2,^40^ and a spreadsheet tool is available for PLCOm2012.^41^ It is possible to integrate these tools into electronic medical records to facilitate calculations, but input information would first need to be verified with the patient. A practical solution may be to use a simplified model or algorithm applied to electronic medical records to initially identify people who are potentially eligible, followed by a more precise assessment of risk using data collected in person or by phone. The goal to automate further the calculation of lung cancer risk and the classification of individual screening eligibility represents an ongoing challenge.

In conclusion, we analysed the performance of lung cancer risk models in three UK cohorts, including the PLCOm2012 and LLPv2 models that are recommended for use in lung screening by the NHS protocol for Targeted Lung Health Checks. We found that the LLPv2 model had worst calibration and classified the lowest proportion of future lung cancer cases as eligible for screening. The LLPv3 model had best calibration (but poor discrimination), while the LCDRAT was best able to identify individuals at high risk of lung cancer. All models strongly over-predicted risk in groups with high SES, raising concerns about exacerbation of disparities in lung cancer screening. Taken together, our results suggest potential revisions to the list of models endorsed by the NHS lung screening protocol and that further work may be needed to ensure that eligibility for lung cancer screening can be defined equitably in the UK population. More generally, they highlight the importance of carefully validating risk prediction models in specific contexts before they are applied in practice.

## Disclaimer

Where authors are identified as personnel of the International Agency for Research on Cancer/World Health Organisation, the authors alone are responsible for the views expressed in this article and they do not necessarily represent the decisions, policy or views of the International Agency for Research on Cancer/World Health Organisation.

## Supplementary information

Supplemental material

## Data Availability

All analysis codes are available upon request. UK Biobank data are available to researchers with an approved request. Access to data from EPIC and the Generations Study requires an application process; further information is available at epic.iarc.fr and breakthroughgenerations.org.uk.
