# Correction: Comparative performance of lung cancer risk models to define lung screening eligibility in the United Kingdom

**DOI:** 10.1038/s41416-021-01436-4

**Published:** 2021-05-17

**Authors:** Hilary A. Robbins, Karine Alcala, Anthony J. Swerdlow, Minouk J. Schoemaker, Nick Wareham, Ruth C. Travis, Philip A. J. Crosbie, Matthew Callister, David R. Baldwin, Rebecca Landy, Mattias Johansson

**Affiliations:** 1 International Agency for Research on Cancer, Lyon, France; 2 The Institute of Cancer Research, London, UK; 3 University of Cambridge, Cambridge, UK; 4 Cancer Epidemiology Unit, Nuffield Department of Population Health, University of Oxford, Oxford, UK; 5 University of Manchester, Manchester, UK; 6 Leeds Teaching Hospitals, Leeds, UK; 7 Nottingham University Hospitals and University of Nottingham, Nottingham, UK; 8 Division of Cancer Epidemiology and Genetics, Department of Health and Human Services, National Cancer Institute, National Institutes of Health, Bethesda, MD USA

**Keywords:** Epidemiology, Lung cancer, Lung cancer, Cancer epidemiology, Cancer screening

Correction to: *British Journal of Cancer* 10.1038/s41416-021-01278-0, published online 12 April 2021

The article Comparative performance of lung cancer risk models to define lung screening eligibility in the United Kingdom, written by Hilary A. Robbins, Karine Alcala, Anthony J. Swerdlow, Minouk J. Schoemaker, Nick Wareham, Ruth C. Travis, Philip A. J. Crosbie, Matthew Callister, David R. Baldwin, Rebecca Landy and Mattias Johansson, was originally published electronically on the publisher’s internet portal on 12th April 2021 without open access. With the author(s)’ decision to opt for Open Choice the copyright of the article changed on 12th February 2021 to © World Health Organization 2021 and the article is forthwith distributed under the terms of the Creative Commons Attribution 3.0 IGO License, which permits use, sharing, adaptation, distribution and reproduction in any medium or format, as long as you give appropriate credit to the World Health Organization, provide a link to the Creative Commons licence and indicate if changes were made.

The use of the World Health Organization’s name, and the use of the World Health Organization’s logo, shall be subject to a separate written licence agreement between the World Health Organization and the user and is not authorized as part of this CC-IGO licence. Note that the link provided below includes additional terms and conditions of the licence.

The images or other third party material in this article are included in the article’s Creative Commons licence, unless indicated otherwise in a credit line to the material. If material is not included in the article’s Creative Commons licence and your intended use is not permitted by statutory regulation or exceeds the permitted use, you will need to obtain permission directly from the copyright holder.

To view a copy of this licence, visit http://creativecommons.org/licenses/by/3.0/igo.

