# Supplementary material for: Comparative performance of lung cancer risk models to define lung screening eligibility in the United Kingdom
Source: Br J Cancer. 2021 Apr 12;124(12):2026–34. doi: 10.1038/s41416-021-01278-0 (PMC8184952; doi:10.1038/s41416-021-01278-0)

**SUPPLEMENT**

**Comparative performance of lung cancer risk models to define lung screening eligibility in the United Kingdom**

Hilary A. Robbins, Karine Alcala, Anthony J. Swerdlow, Minouk J. Schoemaker, Nick Wareham, Ruth C. Travis, Philip A.J. Crosbie, Matthew Callister, David R. Baldwin, Rebecca Landy, and Mattias Johansson

HAR, KA, MJ: International Agency for Research on Cancer, Lyon, France

RL: Division of Cancer Epidemiology and Genetics, National Cancer Institute, National Institutes of Health, Department of Health and Human Services, Bethesda, Maryland, USA

AJS, MJS: The Institute of Cancer Research, London, UK

NW: University of Cambridge, Cambridge, UK

RCT: Cancer Epidemiology Unit, Nuffield Department of Population Health, University of Oxford, Oxford, UK

PAJC: University of Manchester, Manchester, UK

MC: Leeds Teaching Hospitals, Leeds, UK

DRB: Nottingham University Hospitals and University of Nottingham, Nottingham, UK

**Supplementary Methods**

Mapping of UK educational categories to USA categories was necessary in order to validate models that were developed in the USA. For UK Biobank, we mapped less than O-levels to less than grade 12, O-levels/GCSEs or equivalent and CSEs or equivalent to high school graduate; A levels/AS levels or equivalent and other professional qualifications to post-high school, NVQ/HND/HNC or equivalent to some college, and college or university degree to Bachelor’s degree. For EPIC-UK, we mapped primary school and below mapped to less than grade 12, technical school and secondary school to high school graduate, and longer education to Bachelor’s degree. For the Generations Study, only the age left education was provided, in categories. We mapped 10 to 14 years old to less than grade 12, 14 to 17 years old to high school graduate, 17 to 20 years old to some college, and above 20 years old to graduate school.

Values for smoking start-age were tightly clustered with a mode of 17 in all cohorts. We therefore imputed 17 for all missing start-age values (33% in UK Biobank, 10% in EPIC-UK, and 4% in the Generations Study). Subsequently, multiple imputation was used to impute values of smoking quit-years (for former smokers), smoking intensity, education, body mass index, and personal history of cancer. To constrain smoking quit-years so that stopping age could not exceed current age, we calculated the percentage of time since starting smoking spent as quit-time. We imputed this percentage directly, then calculated stopping age and quit-years. Five imputations were performed using the mice package in R with predictive mean matching (version 3.8.0).

Information on first-degree family history of lung cancer, COPD/emphysema, and history of pneumonia was collected only in UK Biobank. Information on asbestos exposure was collected only in EPIC-Cambridge. For each of these variables, a logistic regression model was fit in the cohort that collected the variable, with the outcome as the variable of interest and predictors being age, sex, BMI, years smoked, education, smoking intensity, and race/ethnicity. Parameterizations for continuous variables (BMI, years smoked, and smoking intensity) were adjusted to allow for non-linear relationships as needed. Subsequently, these regression models were used to predict the probability of each variable (e.g., COPD) into the other 3 cohorts, and a random binomial draw with the probability as the mean was used to assign a binary status. Thus, while the probabilities were fixed, the random binomial draw was repeated in each of the 5 imputations.

The LLP, LLPv2, and LLPv3 models include separate terms for family history of lung cancer that depend on the age of diagnosis of the family member (above or below 60 years). This information was not available in any cohort, so age-at-diagnosis was imputed as above 60 years for all participants with a family history.

**Supplementary Table 1**. Model calibration, discrimination, and classification performance for the Hoggart and Liverpool Lung Project (LLP) models.

|  | Hoggart model^28^ | LLP model^19^ |
| --- | --- | --- |
| Calibration (E/O) |  |  |
| UK Biobank | 6.91 (6.00-7.96) | 2.30 (2.18-2.43) |
| EPIC-UK | -- | 2.81 (2.40-3.28) |
| Generations Study | 16.1 (6.69-38.6) | 3.17 (2.42-4.15) |
|  |  |  |
| Discrimination (AUC) |  |  |
| UK Biobank | 0.74 (0.71-0.78) | 0.77 (0.76-0.78) |
| EPIC-UK | -- | 0.82 (0.78-0.85) |
| Generations Study | 0.73 (0.53-0.93) | 0.80 (0.75-0.86) |
|  |  |  |
| Threshold to screen same number as USPSTF 2013 criteria in 3 combined cohorts | 0.9% 1-year risk | 2.4% 5-year risk |
|  |  |  |
| Lung cancer cases classified as screening eligible over 5 years* | 794 (53.9%) | 810 (55.0%) |
|  |  |  |
| Lung cancer deaths classified as screening eligible over 5 years* | 480 (58.1%) | 474 (57.4%) |

*Number of lung cancer cases or deaths classified as screening eligible using the threshold defined above. Percentages represent the percentage of total lung cancer cases or deaths in current and former smokers aged 40-80 that are classified as screening eligible among the 3 combined cohorts. See **Table 3**.

The Hoggart model was developed in EPIC; therefore, we did not evaluate it in EPIC-UK. The time horizon for prediction is 1 year for the Hoggart model and 5 years for the LLP model.

**Supplementary Table 2.** Discrimination estimates for lung cancer risk models in subgroups of UK Biobank, as measured by the area under the ROC curve (AUC).

|  | Area under the ROC curve (AUC) by risk model | | | | | |
| --- | --- | --- | --- | --- | --- | --- |
|  | Bach | LCDRAT | LCRAT | LLPv2 | LLPv3 | PLCOm2012 |
| All participants | 0.80 | 0.82 | 0.81 | 0.77 | 0.78 | 0.79 |
| Sex |  |  |  |  |  |  |
| Male | 0.80 | 0.82 | 0.81 | 0.78 | 0.78 | 0.79 |
| Female | 0.80 | 0.82 | 0.80 | 0.76 | 0.77 | 0.78 |
| Age |  |  |  |  |  |  |
| 40-49 | 0.75 | 0.75 | 0.78 | 0.67 | 0.67 | 0.78 |
| 50-59 | 0.77 | 0.80 | 0.77 | 0.70 | 0.71 | 0.76 |
| 60-69 | 0.76 | 0.78 | 0.76 | 0.73 | 0.73 | 0.75 |
| 70-74 | 0.64 | 0.82 | 0.67 | 0.70 | 0.68 | 0.62 |
| Area-level SES |  |  |  |  |  |  |
| Q1 (highest SES) | 0.79 | 0.84 | 0.79 | 0.77 | 0.79 | 0.76 |
| Q2 | 0.80 | 0.83 | 0.80 | 0.78 | 0.78 | 0.79 |
| Q3 | 0.80 | 0.81 | 0.81 | 0.76 | 0.77 | 0.80 |
| Q4 (lowest SES) | 0.80 | 0.82 | 0.81 | 0.78 | 0.78 | 0.78 |
| Smoking status |  |  |  |  |  |  |
| Current | 0.77 | 0.77 | 0.78 | 0.75 | 0.75 | 0.75 |
| Former | 0.78 | 0.80 | 0.78 | 0.76 | 0.76 | 0.77 |
| Smoking intensity |  |  |  |  |  |  |
| ≤10 CPD | 0.79 | 0.83 | 0.80 | 0.78 | 0.79 | 0.72 |
| 11-29 CPD | 0.80 | 0.82 | 0.80 | 0.77 | 0.77 | 0.81 |
| ≥30 CPD | 0.78 | 0.78 | 0.79 | 0.74 | 0.75 | 0.79 |
| Education |  |  |  |  |  |  |
| Less than secondary | 0.75 | 0.76 | 0.76 | 0.71 | 0.72 | 0.73 |
| Secondary degree | 0.80 | 0.83 | 0.80 | 0.78 | 0.78 | 0.79 |
| Some post-secondary training | 0.80 | 0.82 | 0.80 | 0.74 | 0.76 | 0.77 |
| Some university | 0.75 | 0.80 | 0.77 | 0.72 | 0.77 | 0.75 |
| University graduate | 0.79 | 0.80 | 0.79 | 0.77 | 0.74 | 0.78 |
| Body mass index |  |  |  |  |  |  |
| Underweight | 0.83 | 0.86 | 0.82 | 0.82 | 0.82 | 0.80 |
| Normal weight | 0.83 | 0.84 | 0.84 | 0.79 | 0.79 | 0.80 |
| Overweight | 0.79 | 0.80 | 0.79 | 0.76 | 0.77 | 0.78 |
| Obese | 0.79 | 0.83 | 0.78 | 0.77 | 0.77 | 0.79 |

CPD, cigarettes per day. Estimates are provided for UK Biobank only due to the small size of the other cohorts. The Townsend deprivation index is an area-level measure that is applied to individuals based on their place of residence. Quartiles of the Townsend deprivation index were defined such that UK Biobank participants were divided equally, using the following cutpoints: ‑6.26 (minimum), -3.42, -1.75, 1.21, 11.0 (maximum). Body mass index categories were defined as follows: <18.5 underweight, 18.5-24.9 normal weight, 25-29.9 overweight, and ≥30 obese.

**Supplementary Figure 1.** Calibration estimates for lung cancer risk models in UK Biobank, stratified by quintile of model-predicted risk.


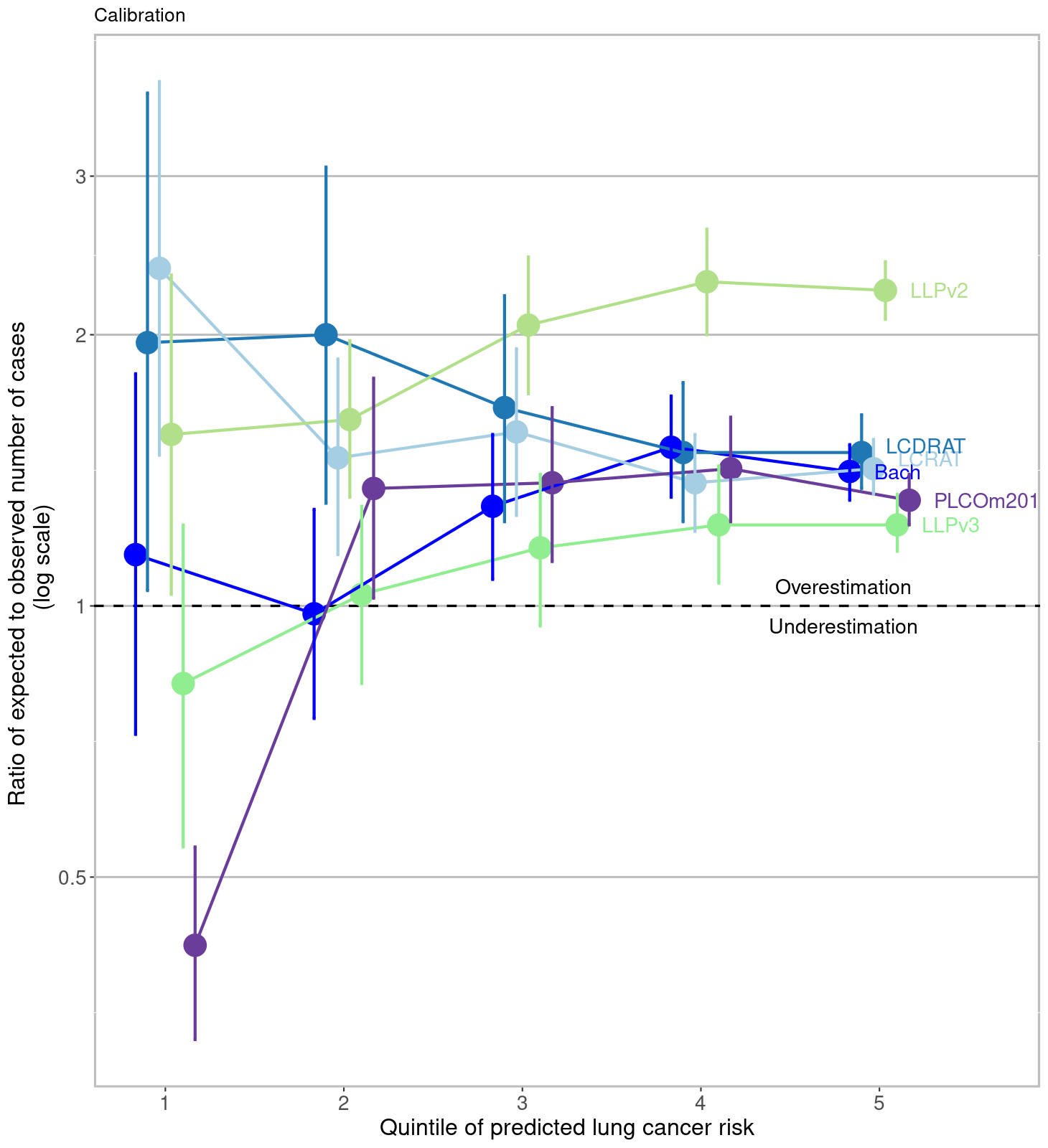


**Supplementary Table 3.** Characteristics of lung cancer cases not identified as screening-eligible (‘missed’) by risk prediction models, among current and former smokers in the combined UK Biobank, EPIC-UK, and Generations Study cohorts. Models are listed in order of their performance for identifying future lung cancer cases in Table 3 (USPSTF 2013).

| **Characteristics of ‘missed’ cases** |  | **Risk prediction model and threshold*** | | | | | |
| --- | --- | --- | --- | --- | --- | --- | --- |
|  | **USPSTF 2013** | **LCDRAT** | **LCRAT** | **PLCOm2012** | **Bach** | **LLPv3** | **LLPv2** |
| **USPSTF 2013 guidelines (age 55-80, at least 30 pack-years, no more than 15 quit-years)** | | | | | | | |
| Threshold* | NA | 0.8%  5-year risk | 1.4%  5-year risk | 1.5%  6-year risk | 1.6%  5-year risk | 1.3%  5-year risk | 2.3%  5-year risk |
| Total, N (%) | 727 (49.3) | 577 (39.1) | 577 (39.1) | 615 (41.7) | 619 (42.0) | 683 (46.3) | 683 (46.3) |
| Age at baseline, median (IQR) | 63 (56-67) | 61 (55-65) | 61 (55-66) | 61 (55-65.5) | 61 (55-65) | 60 (55-65) | 60 (55-65) |
| Age at diagnosis, median (IQR) | 65 (59-69) | 63 (58-68) | 64 (58-68) | 64 (58-68) | 63 (58-68) | 63 (58-67) | 63 (58-68) |
| Female sex, N (%) | 386 (53.1) | 331 (57.4) | 319 (55.3) | 337 (54.8) | 351 (56.7) | 343 (53.7) | 387 (56.7) |
| Former smokers, N (%) | 454 (62.4) | 444 (76.9) | 460 (79.7) | 427 (69.4) | 461 (74.5) | 465 (72.8) | 494 (72.3) |
| Pack-years, median (IQR) | 22 (14-28) | 22 (14-30) | 22 (13-30) | 20 (12-29) | 22 (13-31) | 27 (15-38) | 28 (16-39) |
| Histological type |  |  |  |  |  |  |  |
| Adenocarcinoma, N (%) | 281 (45.4) | 248 (49.3) | 245 (48.7) | 256 (47.9) | 259 (47.9) | 255 (46.6) | 273 (46.3) |
| Squamous cell carcinoma, N (%) | 140 (22.6) | 101 (20.1) | 104 (20.7) | 117 (21.9) | 114 (21.1) | 121 (22.1) | 126 (21.4) |
| Small cell carcinoma, N (%) | 150 (24.2) | 117 (23.3) | 116 (23.1) | 124 (23.2) | 130 (24.0) | 130 (23.8) | 148 (25.1) |
| **USPSTF 2020 guidelines (age 50-80, at least 20 pack-years, no more than 15 quit-years)** | | | | | | | |
|  | **USPSTF 2020** | **LCDRAT** | **LCRAT** | **PLCOm2012** | **Bach** | **LLPv3** | **LLPv2** |
| Threshold* | NA | 0.4%  5-year risk | 0.8%  5-year risk | 0.8%  6-year risk | 0.8%  5-year risk | 0.7%  5-year risk | 1.3%  5-year risk |
| Total, N (%) | 495 (33.6) | 339 (23.0) | 373 (25.3) | 365 (24.8) | 343 (23.3) | 435 (29.5) | 454 (30.8) |
| Age at baseline, median (IQR) | 64 (58-67) | 60 (53-64) | 61 (54-65) | 61 (55-65) | 59 (53-65) | 57 (53-62) | 57 (53-62) |
| Age at diagnosis, median (IQR) | 66 (61-70) | 62 (55-67) | 64 (57-68) | 64 (58-68) | 62 (55-67) | 60 (55-65) | 60 (55-65) |
| Female sex, N (%) | 262 (52.9) | 218 (64.3) | 227 (60.9) | 213 (58.4) | 216 (63.0) | 267 (61.4) | 302 (66.5) |
| Former smokers, N (%) | 376 (76.0) | 281 (82.9) | 321 (86.1) | 274 (75.1) | 283 (82.5) | 303 (69.7) | 306 (67.4) |
| Pack-years, median (IQR) | 17 (10-27) | 18 (10-27) | 18 (10-26) | 14 (9-20) | 16 (9-25) | 25 (14-36) | 25 (14-36) |
| Histological type |  |  |  |  |  |  |  |
| Adenocarcinoma, N (%) | 204 (48.6) | 155 (52.4) | 172 (53.1) | 164 (52.4) | 159 (53.0) | 185 (47.9) | 189 (46.9) |
| Squamous cell carcinoma, N (%) | 88 (21.0) | 48 (16.2) | 53 (16.4) | 52 (16.6) | 47 (15.7) | 76 (19.7) | 78 (19.4) |
| Small cell carcinoma, N (%) | 92 (21.9) | 66 (22.3) | 71 (21.9) | 71 (22.7) | 67 (22.3) | 97 (25.1) | 109 (27.0) |

“Risk” refers to lung cancer death risk for LCDRAT, and to lung cancer risk for all other models. Results are based on a combined dataset which uses a single imputation for missing data. Percentages for histological types do not sum to 100 because other histological types are not shown. Cases with unknown histology are excluded.

*The first set of thresholds is calculated to match the population size selected by the USPSTF 2013 criteria, and the second set is calculated to match the population size selected by the broadened USPSTF 2020 criteria. See Table 3 and footnote.

**Supplementary Figure 2.** Calibration of lung cancer risk models in UK Biobank, restricting to individuals with complete data (N=137,832 or 63% of the full cohort).


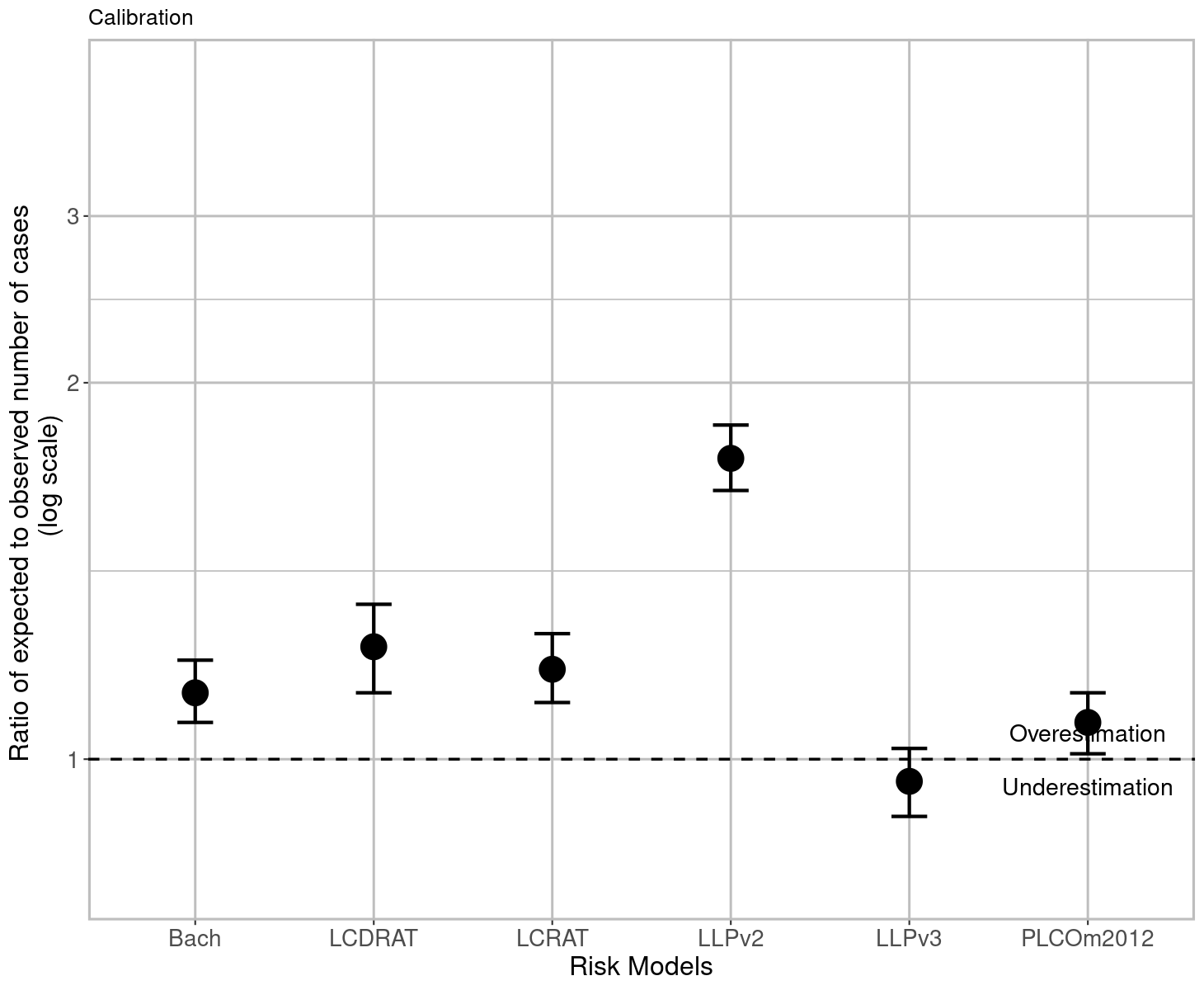


**Supplementary Figure 3.** Discrimination of lung cancer risk models in UK Biobank, restricting to individuals with complete data (N=137,832 or 63% of the full cohort).


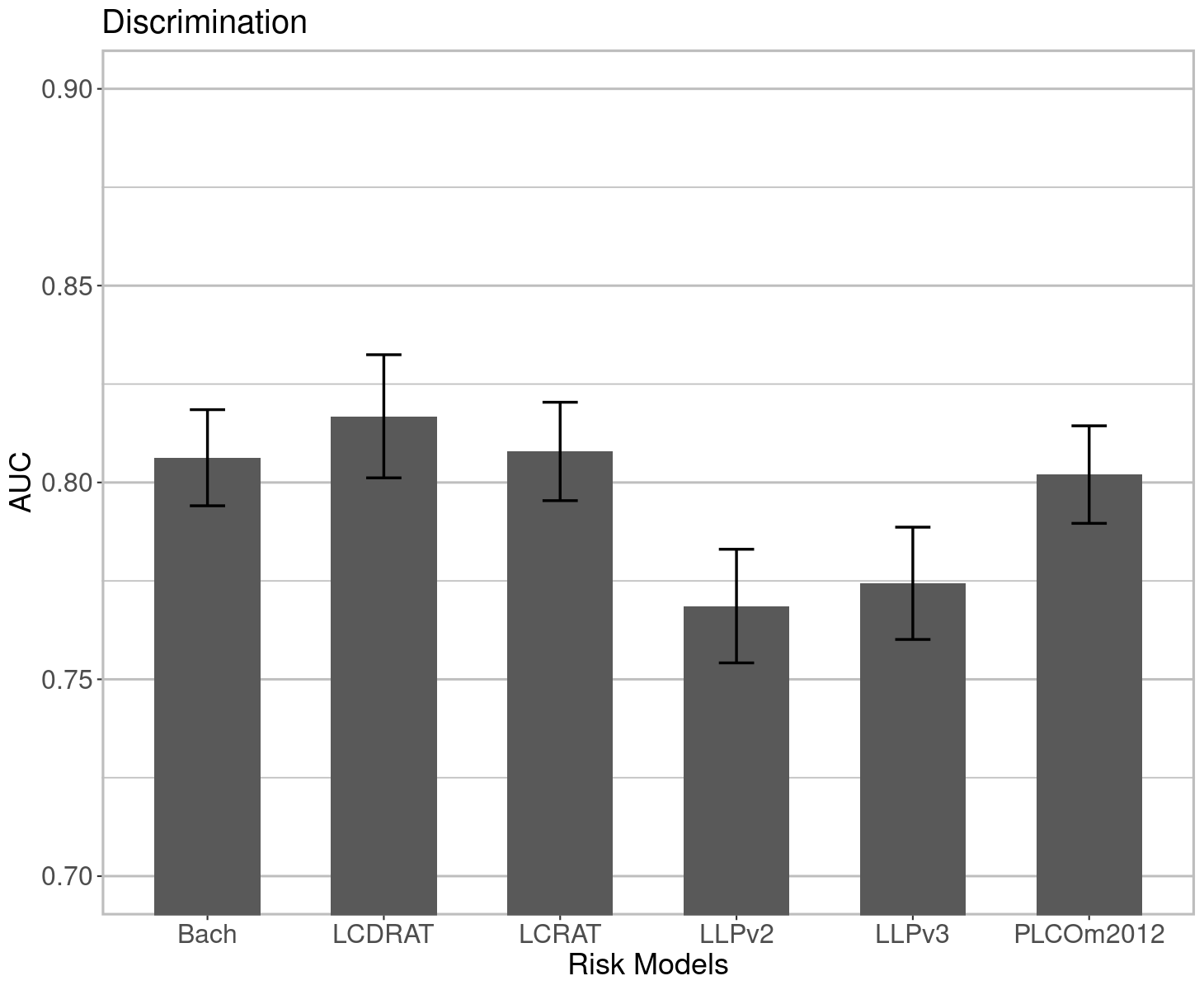

Supplement: Supplementary file 1 — Supplemental material [file 41416_2021_1278_MOESM1_ESM.docx]
